# Genome Sequence of *Salegentibacter salarius* KCTC 12974, Isolated from a Marine Solar Saltern of the Yellow Sea in South Korea

**DOI:** 10.1128/genomeA.01308-16

**Published:** 2016-11-23

**Authors:** Yongle Xu, Jihua Liu, Qiang Zheng, Yanting Liu, Nianzhi Jiao

**Affiliations:** a Institute of Marine Science and Technology, Shandong University, Jinan, Shandong, China; b School of Life Science, Shandong University, Jinan, Shandong, China; c State Key Laboratory of Marine Environmental Sciences, Xiamen University, Xiamen, Fujian, China; d Institute of Marine Microbes and Ecospheres, Xiamen University, Xiamen, Fujian, China

## Abstract

*Salegentibacter salarius* KCTC 12974 is isolated from a marine solar saltern of the Yellow Sea in South Korea. Here, we report the draft genome sequence of *Salegentibacter salarius* KCTC 12974. Various glycoside hydrolase genes in even numbers in the genome reflect the ecological adaption of KCTC 12974 to its habitat.

## GENOME ANNOUNCEMENT

The genus *Salegentibacter* was established by McCammon and Bowman (1) as a member of the family *Flavobacteriaceae*. Members in this genus are Gram-negative, rod-shaped, yellow-pigmented, psychrotolerant, moderately halophilic, and highly halotolerant bacteria, which comprise 10 characterized species to date (1–10). *Salegentibacter salarius* is identified as a member of the genus *Salegentibacter* by Yoon et al. (7). Here, we present the draft genome sequence of *Salegentibacter salarius* KCTC 12974, isolated from a marine solar saltern of the Yellow Sea in South Korea.

Whole-genome sequencing for *Salegentibacter salarius* KCTC 12974, purchased from the Korean Collection for Type Culture (KCTC), was performed using Illumina MiSeq system with average 2 × 250-bp paired-end sequencing kit. A total of ~734 Mbp reads (clean data) were assembled using the Velvet software (version 2.8) (11). The assembled draft genome has a total size of 3,317,048 bp in 58 large contigs, with an average coverage of ~220× and a G+C content of 37.0%. The maximum and average contig size are 305,450 bp and 57,190 bp, respectively. Open reading frames (ORFs) were predicted by combining results from the GeneMark 2.0 gene prediction program (12), Glimmer (13), and the RAST server version 2.0 (14). All predicted ORFs were searched by BLAST against proteins from complete microbial genomes using the NCBI Prokaryotic Genome Annotation Pipeline (15). tRNA sequences in the genome were searched by using tRNAscan-SE (January 2012) (16). rRNA identification was performed by RNAmmer 1.2 software (17). A total of 3,057 protein-coding genes, 40 tRNA-coding genes, and three rRNA genes were predicted in the draft genome.

A detailed genomic inspection of strain KCTC 12974 revealed that the genome contains 170 carbohydrate-active enzyme (CAZyme) genes, as predicted by HMMER3 (18), including 45 genes encoding glycoside hydrolases (GHs), 32 encoding carbohydrate esterases (CEs), and seven encoding polysaccharide lyases (PLs), which are mainly responsible for catalyzing the degradation or modification and potential utilization of carbohydrates. Those 45 GHs in the genome fall into 23 subfamilies in relatively even numbers, catalyzing the hydrolysis or rearrangement of glycosidic bonds of various carbohydrate substrates, including fucose, trehalose, xylose, cellulose, glucoside, inositol, sucrose, galactose, chitin, xylan, starch, fructan, glucan, peptidoglycan, glycosaminoglycan, and others. Various GHs in relatively even numbers are corresponding to the marine feature of strain KCTC 12974’s habitat, which contains diverse carbohydrates existing in low concentrations and reflect the ecological adaption of KCTC 12974 to the marine environment. In addition, the KCTC 12974 genome contains a nitrous oxide reductase gene. The expression of this gene could catalyze the conversion of nitrous oxide to nitrogen and therefore reduce the concentration of nitrous oxide, an efficient ozone-depleting and greenhouse gas in the environment (19, 20).

The genome of strain KCTC 12974 would contribute to the knowledge of marine microbial gene diversity and to a better understanding of the microbial metabolic processes and the element cycling in the marine environment.

### Accession number(s).

The data from this whole-genome shotgun project have been deposited at DDBJ/ENA/GenBank under accession number MJBR00000000. The version described in this paper is the first version, MJBR01000000.
